# Leishmanicidal, Trypanocidal and Antioxidant Activity of Amyrin-Rich Extracts from *Eugenia pyriformis *Cambess

**DOI:** 10.22037/ijpr.2020.113368.14258

**Published:** 2020

**Authors:** João H de Souza, Alexandra Michelon, Fernanda W Banhuk, Izabela V Staffen, Elissandro J Klein, Edson A da Silva, Rafael A Menolli

**Affiliations:** a *Laboratory of Applied Immunology, Center of Medical and Pharmaceutical Sciences, Western Parana State University, Cascavel/PR, Brazil.*; b *Laboratory of Engineering and Environmental Processes, Department of Process and Product Development, State University of Campinas, Campinas/SP, Brazil. *; c *Laboratory of Biotechnological Processes and Separation, Center of Exact and Technological Sciences, Western Parana State University, Toledo/PR, Brazil.*

**Keywords:** Trypanosoma cruzi, Leishmania, Natural extracts, Antioxidant activity, Eugenia, Triterpenoids

## Abstract

Aims: This study aimed to characterize and evaluate leishmanicidal and trypanocidal action as well as cytotoxicity on macrophages and antioxidant ability of extracts, obtained by supercritical CO_2_ and ultrasound-assisted extractions of Uvaia (*Eugenia pyriformis*) leaves. Methods: Leaves from *E. pyriformis *were submitted to supercritical CO_2 _(E1) and ultrasound-assisted (E2) extractions. The characterization of extracts was done using GC-MS and HPLC. *L. amazonensis *(promastigotes) and *T. cruzi *(epimastigotes and trypomastigotes) were treated with crescent concentrations of E1 and E2. After this, parasites were counted and the percentage of inhibition and IC_50_/LC_50_ was calculated. Murine macrophages were treated with both extracts for 48 h and after that, the cellular viability was determined and CC_50_ was calculated. DPPH method was used to determine the antioxidant capacity of both extracts. Results: The results of identification showed a great amount of α and β-amyrin in E1 and E2. Both extracts showed growth inhibition of *L. amazonensis* with an IC_50_ of 5.98 and 9.38 μg/mL to E1 and E2, showing a selectivity index > 30. In trypanocidal tests, an LC_50_ of 16.69 and 7.80 μg/mL (trypomastigotes) and IC_50 _of 5.56 and 34.34 μg/mL (epimastigotes) was reached by E1 and E2. Both extracts showed no toxicity to macrophages and an antioxidant capacity similar to the positive control (tocopherol). Conclusions: This is the first study demonstrating the activity of an amyrin rich-extract against microorganisms that cause Chagas disease and leishmaniasis, as well as its antioxidant capacity, justifying further studies for future *in-vivo* tests.

## Introduction

Chagas disease and leishmaniasis are diseases considered neglected, found mainly in underdeveloped and developing countries. American trypanosomiasis (Chagas disease) is caused by the protozoan *Trypanosoma cruzi, *which is a parasite of mammals, and although transmission through insect vectors is common, infection also occurs due to contaminated food, blood transfusion, placental route, and breast milk, with a great number of infected people in Latin America (1) and recently it became a problem in developed countries, affected by blood transfusion (2). Leishmaniases is a group of diseases caused by protozoa parasites of more than 20 species of *Leishmania*, which are transmitted to humans by the bite of an infected female phlebotomine (3). Parasites of the genus *Leishmania* have a heteroxenous life cycle, alternating between promastigotes (extracellular) and amastigotes (intracellular) forms. Once inside the host, the promastigote forms are internalized by the macrophages, where they differ in immobile amastigote forms constituting the main target of the chemotherapies for leishmaniasis (4).

Despite the significant rates of morbidity and mortality, investment in research, drug production, and transmission control for trypanosomiasis and leishmaniasis are reduced (5). Treatment of trypanosomiasis is effective only in the phase that parasite could be reached in the blood (acute phase), to which two medicines are available: benznidazole and nifurtimox. Benznidazole is a nitroimidazole derivative developed in the 1960s (6) and nifurtimox is a nitrofuran derivative, not been used in Brazil since 1980 due to the emergence of resistant strains in endemic regions (7). Since the 1940s, the first-choice therapy for leishmaniasis has been pentavalent antimonials such as sodium stibogluconate (Pentostan®) and meglumine antimoniate (Glucantime®). These drugs are the main treatments recommended by the WHO (World Health Organization) and are administered parenterally or orally (8). 

*Eugenia pyriformis* Cambess, popularly known in Brazil as Uvaia, has a yellow, edible fruit and can be used to make juices, vinegar, and wine. Pentacyclic triterpenes are distributed throughout the plant kingdom in free form as aglycones or combined forms and have been known to have several biological effects. The triterpenes α-amyrin and β-amyrin and their analogs are commonly found in medicinal plants, being studied due to their chemical and pharmacological properties (9). Since amyrins are present in *E. pyriformis*, the purpose of this research was to determine the antiprotozoal effect, cytotoxicity, antioxidant, and immunoactivity of extracts from Uvaia leaves (10). 

## Experimental

*Preparation and characterization of extracts from E. pyriformis*

Leaves of *E. pyriformis* were collected in a rural property located in Marechal Cândido Rondon (state of Paraná), Brazil. A voucher specimen was identified and deposited in the herbarium (Herbário UNOP) of the Western Paraná State University–UNIOESTE, under the number UNOP 2614. The leaves were dried at room temperature (25 ± 5 °C), in the shade, for 7 days. The extracts were obtained and characterized as described by Klein *et al.,* 2018, using supercritical CO_2 _(pressure 150 bar and temperature 50 °C) and ultrasound-assisted (power 50%, mass/solvent ratio 1:15 g/mL and temperature 50 °C) extraction, using hexane as solvent. Gas chromatography-mass spectrometry analysis (GC-MS) and High-performance liquid chromatography (HPLC) were used for the identification of compounds in extracts. Extracts were diluted in dimethyl sulfoxide (DMSO) to be used in all experiments. Extracts from supercritical and ultrasound-assisted extractions will be named E1 and E2, respectively, throughout this manuscript.

*Determination of free radical scavenging activity (DPPH% assay)*

The free radical scavenging activity was determined by the free radical DPPH (2, 2-diphenyl-1-picrylhydrazyl) method performed according to the protocol described by Arasu *et al*. (2014) with some modifications. The extracts were prepared in concentrations varying from 5 to 175 µg/mL in DMSO and alpha-tocopherol was used as the positive control in the same concentrations of the extracts. Only DMSO was the negative control and DPPH was added to all tubes, and after 30 min of incubation in the dark, the samples were read at 517 nm. The scavenging capacity was calculated using the following equation: 

={1-(abs sample/abs negative control)} × 100

*Anti-Trypanosoma cruzi activity*

Epimastigote forms of *Trypanosoma cruzi* were maintained in Liver Infusion Tryptose (LIT) medium by weekly subcultures and trypomastigote forms of the Y strain of *T. cruzi* were maintained by weekly intraperitoneal passages in mice, from where they were collected by cardiac puncture. Epimastigote (2.5 x 10^5 ^parasites) and trypomastigote forms (1 x 10^5 ^parasites) were subjected to different concentrations of extracts (5, 10, 15, 50, 100, 150, and 175 μg/mL), for 96 and 24 h respectively, after which they were counted in a Neubauer chamber. Only the medium was used as the control, as well as medium-plus DMSO (0.6%), to evaluate its interference. The different concentrations of the compounds were used to determine the inhibitory concentration 50% (IC_50_) and lethal dose 50% (LC_50_). Values of IC_50_ and LC_50_ were determined based on the percentage inhibition of parasite by non-linear regression. The standard drug used as the positive control was benznidazole, which was tested at the same concentrations of the extracts to calculate its IC_50_ on both forms.

*Anti-Leishmania amazonensis activity*

Promastigotes forms of *Leishmania amazonensis* were maintained in RPMI 1640 medium supplemented with 10% fetal bovine serum by weekly subcultures. 1 x 10^5 ^forms were subjected to different concentrations of extracts (5, 10, 15, 50, 100, 150, and 175 μg/mL), for 72 h, after which they were counted in a Neubauer chamber. Only the medium was used as the control, as well as medium-plus DMSO (0.6%), to evaluate its interference. The standard drug used as the positive control was Glucantime®, which was tested at the same concentrations of the extracts. The different concentrations of the compounds and positive control were used to determine the inhibitory concentration 50% (IC_50_). The IC_50_ value was determined based on the percentage inhibition of parasite growth by non-linear regression.

*In-vitro evaluation of activity on murine macrophages*

All legal recommendations of the Brazilian legislation (Law 11.794 Oct. 2008) for animal handling procedures in scientific research were used and this study was approved by the Animal Ethics Committee of Unioeste under number 32/18-CEUA. C57BL/6 mice (6–8 weeks old) were used as peritoneal macrophage donors. Peritoneal macrophages were collected by infusing into the peritoneal cavity of the donors 8–10 mL chilled PBS. The cells were plated in RPMI 1640 culture medium, 5% fetal bovine serum, and antibiotics in 24 or 96-well plates. After 2 h incubation at 37 °C under 5% CO_2_ in a humidified incubator, non-adherent cells were removed by washing twice with warm PBS. Adherent macrophages were incubated for 48 h in the standard medium in the absence (control) or presence of different E1 or E2 concentrations (5, 10, 15, 50, 100, 150, and 175 μg/mL).

Cytotoxicity was evaluated using an MTT (3-(4, 5-Dimethylthiazol-2-yl)-2,5-Diphenyltetrazolium Bromide) reagent, as described by Reilly *et al*. (1998). To measure NO secretion, adherent macrophages (2 × 10^5^ cells/well) were plated in a 96-well plate and incubated in the presence of E1 or E2. LPS (Sigma Chemical Co.) (100 ng/mL) was used as positive control (cell stimulator). After 48 h, NO secretion was indirectly assessed by measuring nitrite concentrations in the culture medium using Griess reaction (13) with modifications. The isolated supernatants were mixed with equal volumes of Griess reagent and incubated at 25 °C for 10 min. Absorbance was measured at 550 nm in a microplate reader. The nitrite concentration was calculated from a standard NaNO_2_ curve (5–100 μM). Results were expressed as μmol per 2 × 10^5^ cells. To determine superoxide production, adherent macrophages were incubated, in 96-well plates, in a standard reaction mixture consisting of HBSS (Hank’s Balanced Salt Solution) containing nitroblue tetrazolium (NBT) (0.02%) and PMA (Phorbol 12-myristate 13-acetate) in the presence or absence of E1 or E2. Control was prepared without extracts and with adequate amounts of DMSO (solvent of extracts). Absorbance was measured at 550 nm after 2 h and the amount of superoxide anion released was demonstrated as previously showed (14).

*Statistical analysis*

All the results are the result of three different experiments conducted in triplicate. The results are presented as mean ± standard error of the mean (SEM) and the data were analyzed statistically by one-way analysis of variance (ANOVA) followed by Tukey’s *posthoc-*test for comparison by GraphPad Prism 6.0 software, at a 95% level of significance. *P* < 0.05 was considered significant. 

## Results

*Characterization and quantification of extracts*

**Figure 1:**
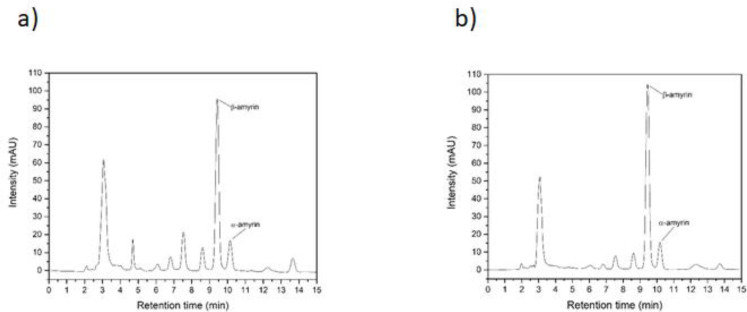
Chromatogram showing extracts from *E. pyriformis *obtained by supercritical CO_2 _(E1) (1a) and ultrasound-assisted (E2) (1b) extractions

The extracts from *E. pyriformis* leaves extracted by supercritical CO_2 _and ultrasound-assisted techniques have been their content showed by Klein *et al.* (2018) (10) and several compounds were detected, as Squalene, Tetratetracontane, Vitamin E, Octadecanal, β-Sitosterol, β-amyrin, and α-amyrin. So, a liquid chromatography was done and showed (Figure 1) great amounts of β-amyrin and α-amyrin. For the extract obtained by supercritical extraction (E1), 17.09 ± 0.27% of α–amyrin and 54.58 ± 0.09% of β–amyrin (mean ± SD of three dosages) were quantified, very close to that determined by HPLC for ultrasound-assisted extract (E2), with 14.31 ± 0.36% (α–amyrin) and 62.72 ± 0.50% (β–amyrin). 

*Antioxidant activity*

(Table 1( lists the results of the DPPH test performed with *E. pyriformis* extracts. Analyzing the extracts with their respective controls, we verified that with increasing concentration of the extract, the percentage of the antioxidant activity also increased, demonstrated by the percentage values. It is also verified that the positive control had higher values from the concentration of 10 μg/mL in E1, and the first concentration in E2, however, no significant differences between the positive control and its respective concentration in the extract were detected, demonstrating its antioxidant capacity.

**Figure 2:**
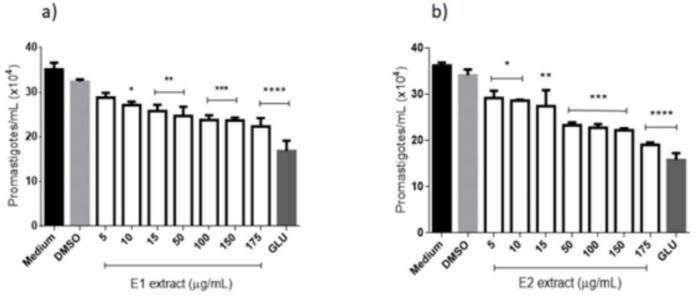
Anti-*Leishmania* (promastigotes forms) activity of extracts from *E. pyriformis* obtained by supercritical CO_2_ (E1) (a) and ultrasound-assisted (E2) (b) extractions, after 96 h *, **, *** and **** indicate *p* < 0.05, *p* < 0.01, *p* < 0.001, and *p* < 0.0001, respectively, relative to the medium. GLU-Glucantime® (positive control–300 µg/mL)

*Anti-protozoa activity*

(Figure 2a) illustrates the promastigotes of *L. amazonensis *treated with the extract obtained by supercritical extraction (E1), which caused a decrease in the parasite number at all concentrations tested. Significant differences were found between negative control (medium) and all tested concentrations of E1, except for 10 μg/mL, after 96 h of contact. Positive control (Glucantime®) at 175 μg/mL showed no significant differences from E1 at concentrations of 150 and 175 μg/mL. (Figure 2b) shows the action of E2 extract against culture forms of *L. amazonensis *and a gradual reduction in the number of parasites with increasing concentration of the extract. All the tested concentrations showed significant differences from the negative control and Glucantime® at 175 μg/mL did not decrease significantly more than E2 at 150 and 175 μg/mL. The IC_50_ was calculated for extracts and E1 and E2 reached 5.98 µg/mL and 9.38 µg/mL, respectively, the values more expressive than that reached by Glucantime® (20.47 µg/mL) (Table 2).

**Figure 3:**
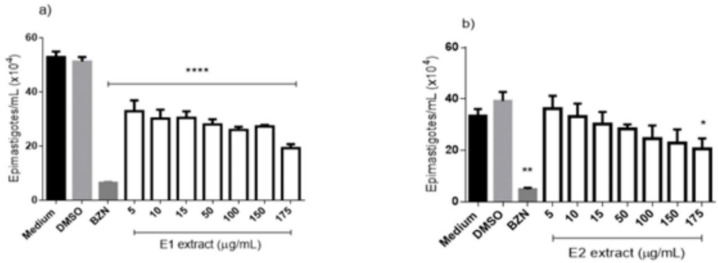
Anti-*Trypanosoma cruzi* (epimastigotes forms) activity of extracts from *E. pyriformis* obtained by supercritical CO_2_ (E1) (a) and ultrasound-assisted (E2) (b) extractions, after 96 h. *, ** and **** indicate *p* < 0.05, *p* < 0.01, and *p* < 0.0001, respectively, relative to the medium. BZN–Benznidazole (positive control–50 µg/mL)

The activity against epimastigotes forms of *T. cruzi* is shown in (Figure 3) and demonstrates that all tested concentrations of E1 extract (Figure 3a), after 96h, differ significantly from the negative control, reaching an inhibition rate of 63.5% at the concentration of 175 μg/mL. The E2 extract (Figure 3b) obtained a worst performance than E1, reaching a significant difference from the negative control only in the higher concentration (175 μg/mL), which demonstrated an inhibition rate of 38.7%. The IC_50_ to the extracts showed values of 5.56 and 34.34 to E1 and E2, respectively. The benznidazole reached an inhibition rate of 93.3% at 50 μg/mL and an IC_50_ of 3.13 μg/mL (Table 2).

**Figure 4:**
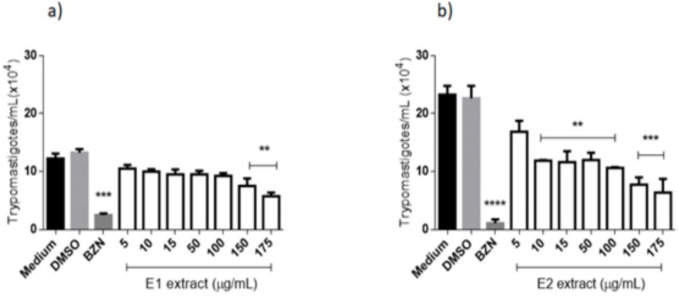
Anti-*Trypanosoma cruzi* (trypomastigotes forms) activity of extracts from *E. pyriformis* obtained by supercritical CO_2_ (E1) (a) and ultrasound-assisted (E2) (b) extractions, after 24 h **, *** and **** indicate *p* < 0.01, *p* < 0.001, and *p* < 0.0001, respectively, relative to the medium. BZN–Benznidazole (positive control–50 µg/mL)

Trypanocidal activity of the extracts from *E. pyriformis* against *T. cruzi* infecting forms was also tested, and the action of E1 on trypomastigote can be verified in (Figure 4a). After 24 h, the negative control presented statistical differences from the concentration of 175 μg/mL and the positive control (benznidazole 50 μg/mL). This extract obtained an LC_50_ value of 16.69 μg/mL, exhibiting moderate activity against *T. cruzi* trypomastigote when compared to benznidazole (LC_50_ = 7.26 μg/mL). For E2 (Figure 4b), a significant difference was verified when comparing the negative control to all tested concentrations, except for 5 μg/mL, after 24 h of contact. Benznidazole at 50 μg/mL did not present a statistical difference from E1 at 175 μg/mL, indicating activity against the parasite. LC_50_ obtained by E2 (7.80 μg/mL) was very similar to that reached by benznidazole, demonstrating a high activity of this extract against infective forms of *T. cruzi*.

*Cellular viability*

**Figure 5.**
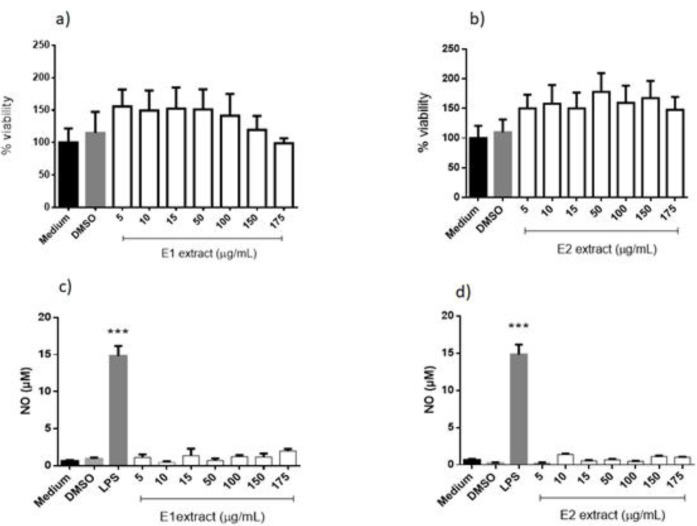
Cell viability (a, b) and nitric oxide (NO) production (c, d) of murine peritoneal macrophages treated with extracts *Eugenia pyriformis* obtained by supercritical CO_2_ (a, c) and ultrasound-assisted (b, d) extractions. *** indicate *p* < 0.001, relative to the medium

The extracts of Uvaia, specifically the E1 extract, had no interference in the viability of the macrophages, showing low or null toxicity to the cells (Figura 5a), still was showed here that the solvent present in the extracts, DMSO, also did not affect the viability of the macrophages, being similar to the cells that was incubated only in medium.

We also evaluated if the extracts could have a selectivity action against the parasite and yet not kill or interfere in the viability of the host macrophages, to do so, we calculated the selectivity index (SI) considering the CC_50 _and IC_50_ values. Here the E1 extracts had a SI of 50.1 to *L. amazonensis *parasites whereas to the *T. cruzi *epimastigotes the SI was 53.96 and to the *T. cruzi *trypomastigotes forms the SI was 17.97 (Table 2). A safety SI is considered when the value is higher thant 10 (15). 

The other extract, E2, the one obtained by ultrasound-assisted extraction from *E. pyriformis*, also did not affect the viability of the macrophages, achieving rates close to the cells that were treated only with the medium (negative control). The data also shows that the DMSO presented in all the extracts did not interfere in the survival of the cells, again. 

Thus, when calculating the SI of the E2 extract, one could check that this extract had a better SI than the one produced by the drug Glucantime®. Altogether the E2 presented a SI of over 31.98 against promastigote of *Leishmania* and over 38.46 against promastigotes of *T. cruzi*, while Glucantime® achieved a SI of over 14.66 and benznidazole had a SI over 41.32. The SI of this extract against the epimastigotes forms of *T. cruzi* was only over 8.74, a number that is not considered satisfactory to a SI index.

*Effects on murine macrophages*

(Figures 5c and 5d) show that murine macrophages treated with E1 and E2 were not able to produce NO, different from those treated with LPS (positive control) and very similar to medium and DMSO controls. Superoxide production by macrophages treated with E1 and E2 was not altered (Supplementary file Figure S1), demonstrating the same behavior as the controls, even in the presence of PMA, without interfering in the production process of reactive oxygen species.

## Discussion

The presence of amyrins in plants has been detected from different sources as resins, bark, pollen, or leaves. They were extracted isolated or in a mixture, which seems to be less common, with variations in the percentage of both (16). When using solvent and differente methods to extract the secondary metabolites from the plants, it is usual to extract more than one substance together (17) and this is what happened here, which extracted amyrins but with other components together.

The activity of amyrins has been demonstrated by several studies, and some fractions with different metabolites tested by Frankenberger *et al.* (2018) (18) revealed an interesting antiparasitic effect on intracellular amastigotes of *L. amazonensis* and trypomastigotes of *T. cruzi*. The antiparasitic activities of the fractions were better than those of the semi-synthetic triterpenes (prepared from three different reactions of α-amyrin oxidation) and α-amyrin. This latter result suggested a synergistic contribution of the fraction constituents. Results presented by Otuki *et al.* (2005) (19) showed that the systemic administration of the triterpenes α and β-amyrin via the spinal and supraspinal pathways in mice produces pronounced antinociception and is dose-dependent. Also, this effect appears to be related to its ability to interfere with the Protein kinase C and A pathways.

Amyrins have demonstrated antioxidant activity (20) when applied pure or in mixture with other compounds, which is common in plant extracts. DPPH is a stable nitrogen-centered free radical, and its color changes from violet to yellow when it is reduced by either the process of hydrogen or electron-donation. Substances that can perform this reaction can be considered as antioxidants and radical scavengers, and values obtained by E1 and E2, very similar to α-tocopherol (a powerful antioxidant) show the ability of these extracts in this direction. Victoria *et al.* (2012) (21) showed that the essential oil of the leaves of *Eugenia uniflora*, the same genus of *E. pyriformis*, obtained an antioxidant activity in three different tests - DPPH method, 20-azino-bis-ethylbenzthiazoline-6-sulfonic acid (ABTS), and the Ferric Reducing Antioxidant Power (FRAP).

A fraction containing terpenoids were tested against *L. donovani *promastigotes and showed significant growth inhibition, presenting an IC_50_ = 18.75 μg/mL (22), similar to that obtained by E2 but higher than that obtained by E1. This can be due to some metabolites found in different plants providing different results even then the extraction process is the same. In some cases, the activity of the extracts is remarkable, although generally not as successful as the isolated compounds (23).

Studies with the action of amyrins on *Leishmania* are scarce, but other species of the genus *Eugenia *(*E. uniflora* and *E. umbeliflora*) have their extracts tested against *L. amazonensis *and showed antileishmanial activity against promastigotes, but the essential oil of the bark of *E. uniflora* was inactive against promastigotes of *L. donovani* (24).

Mwangi *et al.* (2010) (25) have revealed that α-amyrin or β-amyrin exhibits low or no activity, with an IC_50_ ≥ 30 μg/mL, against trypomastigote forms of *T. cruzi*, when used isolated. Other authors have investigated the trypanocidal activity of amyrins and have shown that pure compounds, including α and β-amyrin, are inactive (26). Confirming that, the use of mixtures of triterpenes may present better biological activities than their isolated compounds, which justifies the use of α and β–amyrin together (27, 28). So, the activity obtained in this study can be a synergistic effect of these substances when together or combined with the other compounds in the extracts from the plants. No studies were found showing amyrins against epimastigotes of *T. cruzi*, but a hydroalcoholic extract from *Eugenia jambolana *showed an IC_50 _of 5 µg/mL against epimastigotes from *T. cruzi *(29), and despite not being characterized, demonstrated the potential of the genera, as evidenced by the E1 and E2 from *E. pyriformis*. 

Lima *et al.* (2015) (30) demonstrated the leishmanicidal activity of a diterpene against promastigotes and amastigotes of *L. amazonensis*, with SI of 625.0 μg/mL, demonstrating that this molecule did not cause cytotoxic effects on macrophages and was highly selective for the parasite. Also, it is important to conduct toxicity tests on macrophages not only to determine SI but also because *Leishmania* parasites inhabit these cells (31). 

The α and β-amyrin have low cytotoxicity in normal mammalian cells (32, 33), which confirms the data obtained by this study, in which amyrin-rich extracts of *E. pyriformis* did not show aggression to macrophages *in-vitro*. Besides, these compounds were also able to differentiate tumor cells from healthy human cells, demonstrating selectivity in another situation (34). 

Nitric oxide (NO) is secreted at high concentrations by macrophages as part of its mechanism of toxicity against microorganisms. In humans, the microbicidal activity of NO released by macrophages is induced by lipopolysaccharides (LPS) and by some cytokines such as interferon (IFN-γ). It is also generated after activation of macrophage by IFN-γ and tumor necrosis factor (TNF) and, it is very important to eliminate the intracellular forms of *Leishmania* (35). This study agreed with previous studies, which point out both α and β-amyrins as anti-inflammatory, antinociceptive, and analgesic substances, being able to revert the cellular infiltrate in a visceral or periodontal inflammatory process and inhibit the production of NO, by inhibition of inducible NO synthase (iNOs) (36–39). The activity of NO responsible for the destruction of intracellular forms of *Leishmania* and *T. cruzi* would be compromised by the presence of amyrins unless they exert direct intracellular destruction without the use of the macrophage phagocytic capacity. This suggests the continuity of this study with the intracellular forms of these parasites.

So, the extracts from *E. pyriformis *proved to contain a great amount of α and β-amyrins, demonstrating antioxidant, leishmanicidal, and trypanocidal activities but were not capable to stimulate immune cells, as demonstrated herein. Further studies should be conducted with extracts of *E. pyriformis* to demonstrate their abilities against intracellular forms of the parasites and *in-vivo* models.

**Table 1:** Percentage (± SD) of antioxidant activity (DPPH method) of extracts obtained from *E. pyriformis* by supercritical CO_2_ (E1) and ultrasound-assisted (E2) extractions. Positive controls were α-tocopherol

**Concentration (µg/mL)**	**E1 (AA%)**	**Positive control (AA%)**	**E2 (AA%)**	**Positive control (AA%)**
5	12.27 ± 3.41	12.73 ± 3.45	9.45 ± 2.13	12.04 ± 2.21
10	16.02 ± 0.81	20.34 ± 3.16	10.52 ± 3.52	14.45 ± 2.06
15	17.99 ± 3.17	24.85 ± 2.38	11.79 ± 2.22	21.86 ± 1.40
50	22.81 ± 0.83	27.34 ± 0.64	18.57 ± 2.28	23.49 ± 1.89
100	25.73 ± 3.21	33.69 ± 3.66	21.76 ± 2.73	26.17 ± 0.12
150	27.60 ± 3.31	37.30 ± 3.88	22.01 ± 2.34	28.63 ± 3.22
175	28.3 7± 3.07	38.06 ± 0.53	23.91 ± 1.70	32.29 ± 2.24

AA–antioxidant activity

**Table 2.** IC_50_, LC_50, _and CC_50_ data obtained from epimastigotes, trypomastigotes, and promastigotes forms and murine macrophages treated with E1 and E2 extracts

**Extracts**	***T. cruzi *** **Epimastigotes (96 h) IC**_50 _ **(µg/mL)**	***T. cruzi *** **Trypomastigotes (24 h) LC**_50 _ **(µg/mL)**	***L. amazonensis *** **Promastigotes (96 h) IC**_50 _ **(µg/mL)**	**Macrophages CC**_50 _ **(µg/mL)**	**SI **_(epi)_	**SI **_(trypo)_	**SI **_(pro)_
E1	5.56	16.69	5.98	> 300	> 53.96	> 17.97	> 50.17
E2	34.34	7.80	9.38	> 300	> 8.74	> 38.46	> 31.98
GLU	-	-	20.47	> 300	-	-	> 14.66
BZN	3.13	7.26	-	> 300	> 95.85	> 41.32	-

E1: Extract obtained by supercritical CO_2 _extraction from *E. pyriformis *leaves; E2: Extract obtained by ultrasound-assisted extraction from *E. pyriformis *leaves; BZN: benznidazole (reference drug control to *T. cruzi*); GLU: Glucantime® (reference drug control to *Leishmania)*. SI (selectivity index): CC_50_/IC_50 _or LC_50._

## Supplementary Materials

Supplement
