# Supplementary material for: Leishmanicidal, Trypanocidal and Antioxidant Activity of Amyrin-Rich Extracts from Eugenia pyriformis Cambess
Source: Iran J Pharm Res. 2020 Autumn;19(4):343–53. doi: 10.22037/ijpr.2020.113368.14258 (PMC8019871; doi:10.22037/ijpr.2020.113368.14258)
Supplement: Supplement [file ijpr-19-343-s001.pdf]

Supplementary Materials for

Leishmanicidal, Trypanocidal And Antioxidant Activity of Amyrin-Rich Extracts from *Eugenia Pyriformis* Cambess

João H de Souza, Alexandra Michelon, Fernanda W Banhuk, Izabela V. Staffen, Elissandro J. Klein, Edson A. da Silva and Rafael A Menolli*

*To whom correspondence should be addressed. E-mail: rafael.menolli@unioeste.br

Volume 19, Issue 4 (Autumn 2020)

This PDF file includes:
Figure S1

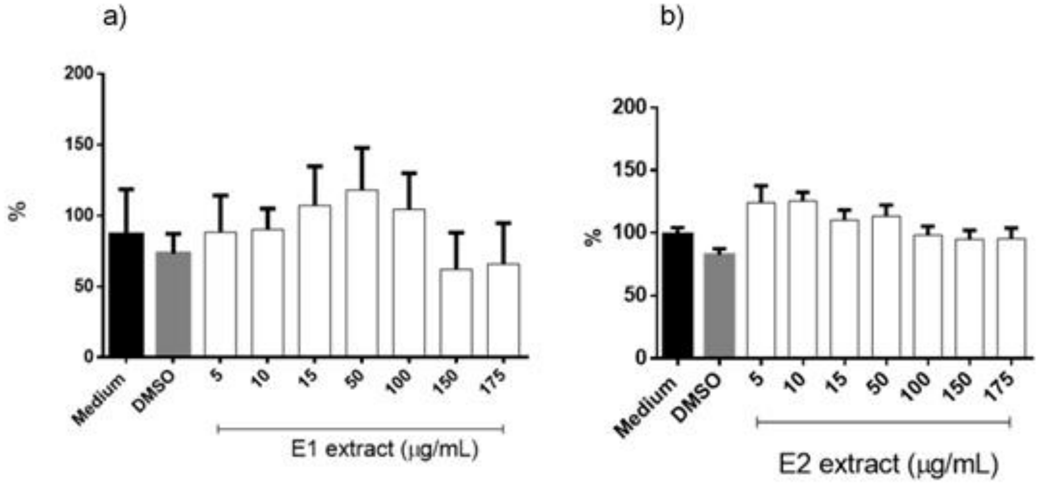

Figure S1. Effects of supercritical CO_2 (E1) (a) and ultrasound-assisted (E2) (b) extracts from *E. pyriformis* on superoxide anion production by peritoneal murine macrophages. The data are expressed in percentages about the medium, which was considered 100%. The values shown are the mean ± SEM of three independent experiments, each performed in triplicate. Control corresponds to cells treated with culture medium ou culture medium plus DMSO.
